# A glance at the hidden diversity of Rubisco and CO_2_-concentrating mechanisms in cyanobacteria

**DOI:** 10.1093/plphys/kiag286

**Published:** 2026-05-22

**Authors:** Matheus E Bianconi

**Affiliations:** Assistant Features Editor, Plant Physiology, American Society of Plant Biologists, Rockville, MD, United States; Laboratoire de Recherche en Sciences Végétales (LRSV), Université de Toulouse, CNRS, UPS, Toulouse INP, Castanet-Tolosan, France

Cyanobacteria are photosynthetic prokaryotes ubiquitous across marine, freshwater, and terrestrial habitats worldwide. The group emerged early during prokaryote diversification, around 2.5 to 3 billion years ago (Schirrmeister et al 2013), and played a central role in shaping life as we know it. Oxygen released by their photosynthetic activity contributed to the Great Oxidation Event, creating the conditions for early eukaryotic life to establish. The photosynthetic machinery of the green eukaryotic lineage (green algae and land plants) is descended from a single cyanobacterial ancestor (Ponce-Toledo et al 2017), which was incorporated via an endosymbiotic event by an early eukaryotic lineage around 1.5 billion years ago. Such a long history of evolutionary diversification is reflected in the broad range of morphological and metabolic adaptations across extant cyanobacteria (Stanier and Cohen-Bazire 1977). Unraveling the mechanistic basis of key cyanobacterial traits, particularly those related to photosynthesis and N_2_ fixation, has reinforced scientific interest in cyanobacteria over the past decades and could inform synthetic biology efforts (Rae et al 2017).

As in the green eukaryotic lineage, CO_2_ is fixed in cyanobacteria by the enzyme ribulose 1,5-bisphosphate carboxylase/oxygenase (Rubisco). A well-known feature of Rubisco is that CO_2_ and O_2_ compete at the active site of the enzyme and that O_2_ fixation triggers photorespiration, a metabolic pathway that results in net CO_2_ loss. This wasteful reaction had little impact in the CO_2_-rich, anoxygenic atmosphere in which Rubisco evolved, but this changed with the rise of atmospheric O_2_ and decline of CO_2_ over geological time. Despite billions of years of action of natural selection, a Rubisco form free from its oxygenase activity probably never evolved, likely due to the intrinsic catalytic constraints of the enzyme (Tcherkez et al 2006). Instead, “workaround” solutions that increase the relative concentration of CO_2_ near Rubisco emerged, and these can virtually suppress the enzyme's oxygenase activity. Notable examples of such CO_2_-concentrating mechanisms (CCMs) are C_4_ and Crassulacean acid metabolism photosynthesis in land plants, which are important adaptations to low CO_2_ availability. Cyanobacteria also evolved CCMs, and these have been optimized over billions of years of evolution compared to the more recently evolved CCMs of land plants. The functional diversity of Rubisco and CCMs in cyanobacteria, however, remains largely unexplored. In an article in the *Plant Physiology* focus collection on algae and aquatic plants, Aguiló-Nicolau and colleagues (2026) conducted a detailed study on Rubisco kinetics and CCMs in five cyanobacterial strains from disparate lineages, broadening our understanding of Rubisco's biochemistry in the group and providing new insights into the factors determining CCM efficiency.

Rubisco's catalytic efficiency is commonly described by two parameters: the carboxylation turnover rate ($k_{\mathrm{cat}}^{c}$) and the enzyme's specificity for CO_2_ relative to O_2_ (S_c/o_). Rubisco evolved a high specificity to CO_2_ across photosynthetic lineages, but it is often perceived as a slow catalyst, with turnover rates mostly below 10 carboxylation reactions per second, which is one or more orders of magnitude lower than enzymes catalyzing comparable reactions (Bathellier et al 2018). Aguiló-Nicolau et al (2026) observed that kinetic properties were not homogeneous across their cyanobacterial strains. For example, $k_{\mathrm{cat}}^{c}$ ranged from ∼ 8 to 12 s^−1^ and S_c/o_ from to ∼ 48 to 74 mol mol^−1^, which are values within the range previously reported for land plants and other photosynthetic organisms (Bouvier et al 2024). They also tested whether closely related taxa tend to exhibit similar kinetic traits but detected no significant phylogenetic signal for any of the parameters examined. This suggests that the diversification of Rubisco kinetics is not strongly constrained by ancestral states within cyanobacteria lineages, at least not across such a deep evolutionary scale (cf. Bouvier et al 2021).

Cyanobacteria are among the most morphologically diverse prokaryotes, ranging from uni- to multicellular filamentous forms, and bear photosynthetic machinery with widely varied compositions. The strains selected by Aguiló-Nicolau and colleagues (2026) encompass a range of morphologies, from free living coccoid cells (eg, *Cyanobacterium aponinum*, Fig. 1a, and *Synechococcus* sp., Fig. 1e) to filamentous forms (*Oscillatoria acuminata*; Fig. 1c). Using transmission electron microscopy, the authors observed that thylakoid organization was also variable (Fig. 1). While some strains have an extensive network of thylakoids, *Gloeobacter violaceous* PCC7421 (Fig. 1b) completely lacks it. This strain belongs to the Gloeobacterales order, known for lacking thylakoids and conducting the light-dependent reactions of photosynthesis in the cytoplasmic membrane.

**Figure 1:**
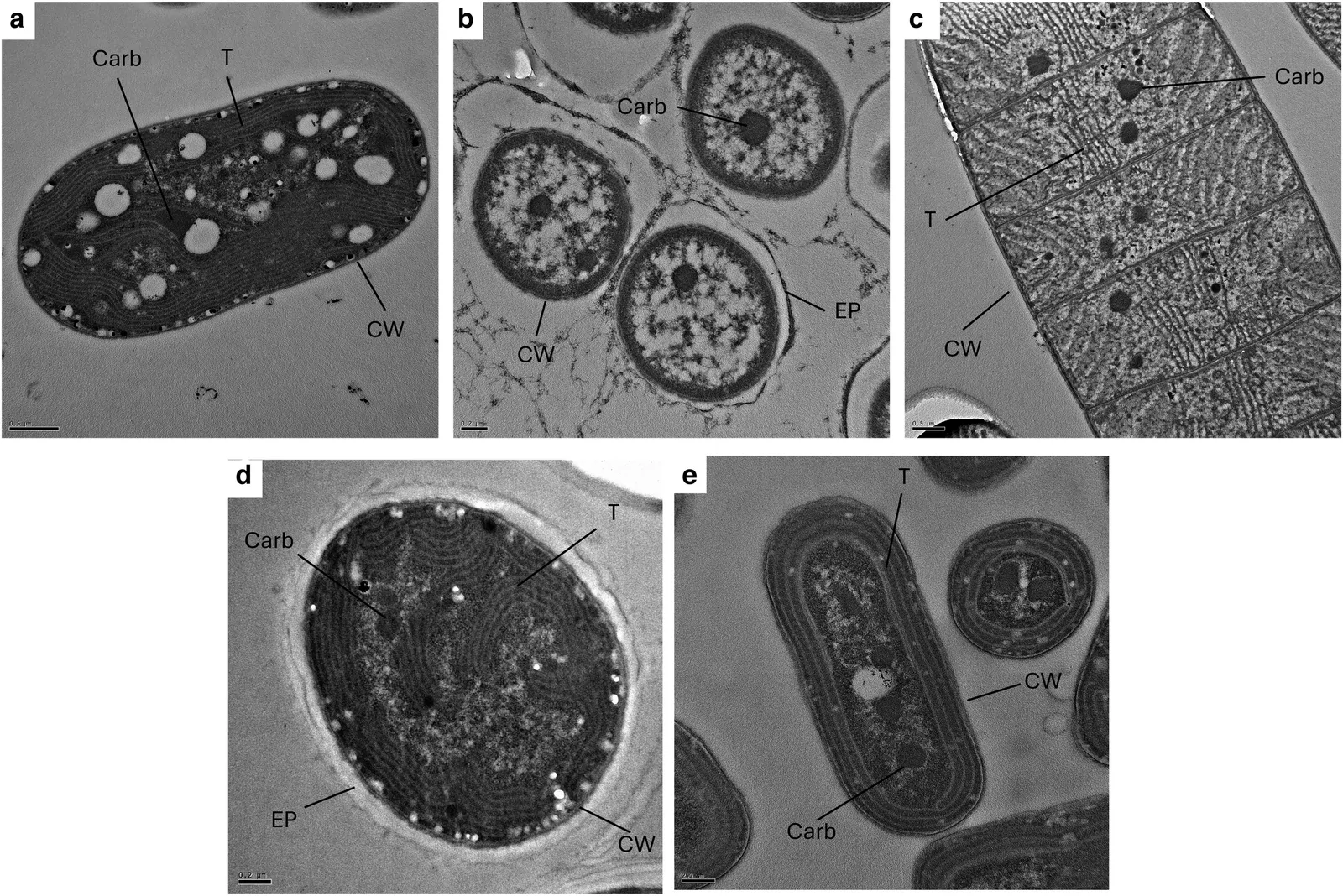
Linking structure and function of CO_2_-concentrating mechanisms across the diversity of cyanobacteria. Transmission electron micrographs of the cyanobacteria strains studied by Aguiló-Nicolau and colleagues (2026). a) *Cyanobacterium aponinum* PCC10605 (Chroococcales), b) *Gloeobacter violaceous* PCC7421 (Gloeobacterales), c) *Oscillatoria acuminata* PCC 6304 (Oscillatoriales), d) *Chroococcidiopsis thermalis* KOMAREK 1964/111 (Chroococcidiopsidales), and e) *Synechococcus* sp. PCC6301 (Synechococcales). Carb, carboxysome; CW, cell wall; EP, exopolysaccharide; T, thylakoid membrane. Scale bars: (a, c) 500 nm; (b, d, e) 200 nm. Micrographs reproduced from Aguiló-Nicolau et al. (2026).

This structural diversity is accompanied by variation in CCM components. The basic CCM architecture in cyanobacteria comprises dedicated membrane transporters that actively import inorganic carbon (mainly as bicarbonate, $\mathrm{HC}O_{3}^{-}$) into the cytoplasm, together with carbonic anhydrases (CAs) that convert $\mathrm{HC}O_{3}^{-}$ to CO_2_ at the site of Rubisco. CAs are often encapsulated together with Rubisco within specialized microcompartments known as carboxysomes, which are ubiquitous in cyanobacteria (Kerfeld and Melnicki 2016). With such an arrangement, CO_2_ concentration can build up to high levels around Rubisco, suppressing the enzyme's oxygenase activity. By coupling biochemical and physiological measurements to quantify carbon fixation across the strains, the authors observed that CCM effectiveness is strongly dependent on CA activity and partially explained by the cell volume occupied by carboxysomes.

The study by Aguiló-Nicolau and colleagues (2026) explores the diversity of Rubisco kinetic parameters and CCM-related traits across cyanobacteria. Given the deep evolutionary divergence among extant cyanobacterial lineages, even within the major orders (Schirrmeister et al 2013), it is possible that a significant fraction of the Rubisco kinetic space in cyanobacteria remains unknown. Studies that integrate functional genomics and physiology with phylogenetics could help disentangle how individual CCM components contribute to carbon fixation efficiency in these organisms. Genomic studies using a pangenome framework can be particularly informative, as sampling closely related strains across environmental gradients can reveal how CCM architecture and ecology are correlated. Beyond their evolutionary significance, filling these fundamental gaps may help design operational single-cell CCMs that could be incorporated into crops to boost photosynthetic efficiency.

## Recent related articles in Plant Physiology

- Cheng et al (2024) deciphered the structure of a chaperone protein involved in carboxysome assembly in the cyanobacterium *Nostoc* sp. PCC7120 and proposed a model of how shell proteins and Rubisco interact during the formation and maturation of carboxysomes.
- Sun et al (2025) conducted a high-resolution study on the ultrastructure of carboxysomes in the freshwater cyanobacterium *Synechococcus elongatus* PCC7942, revealing how Rubisco enzymes are arranged within carboxysomes.

## Data Availability

No new data were generated or analysed in support of this News and Views article.
